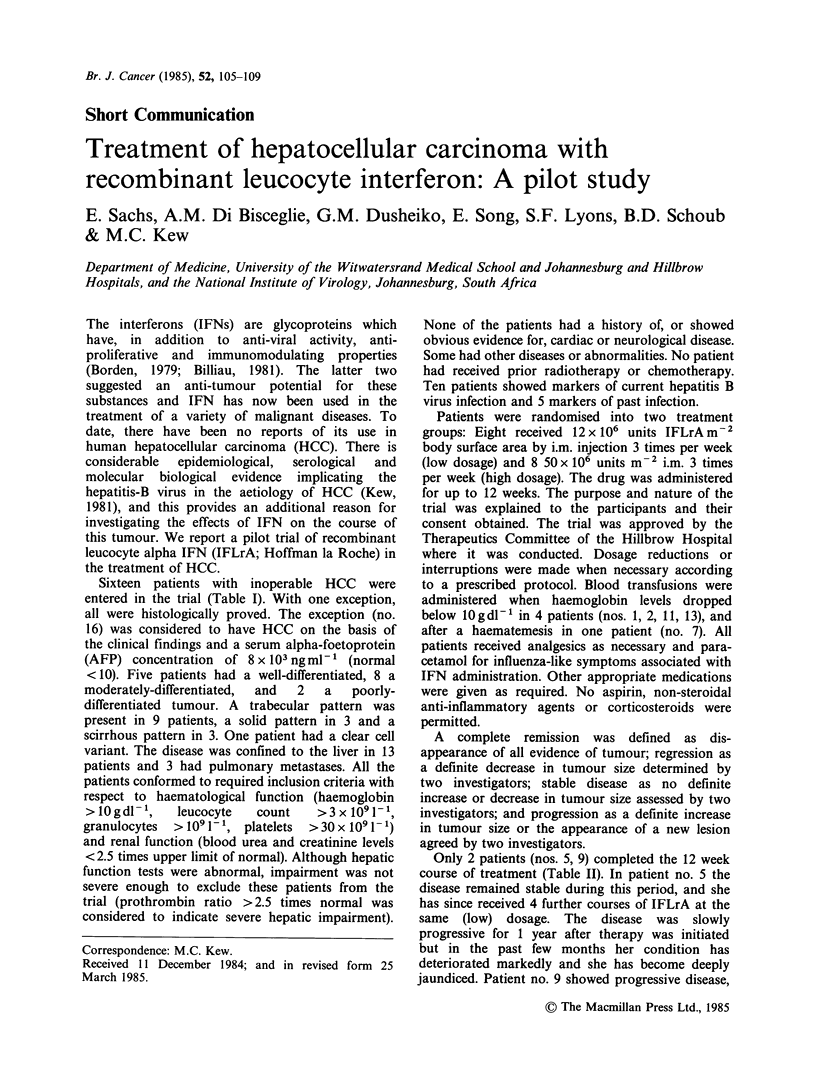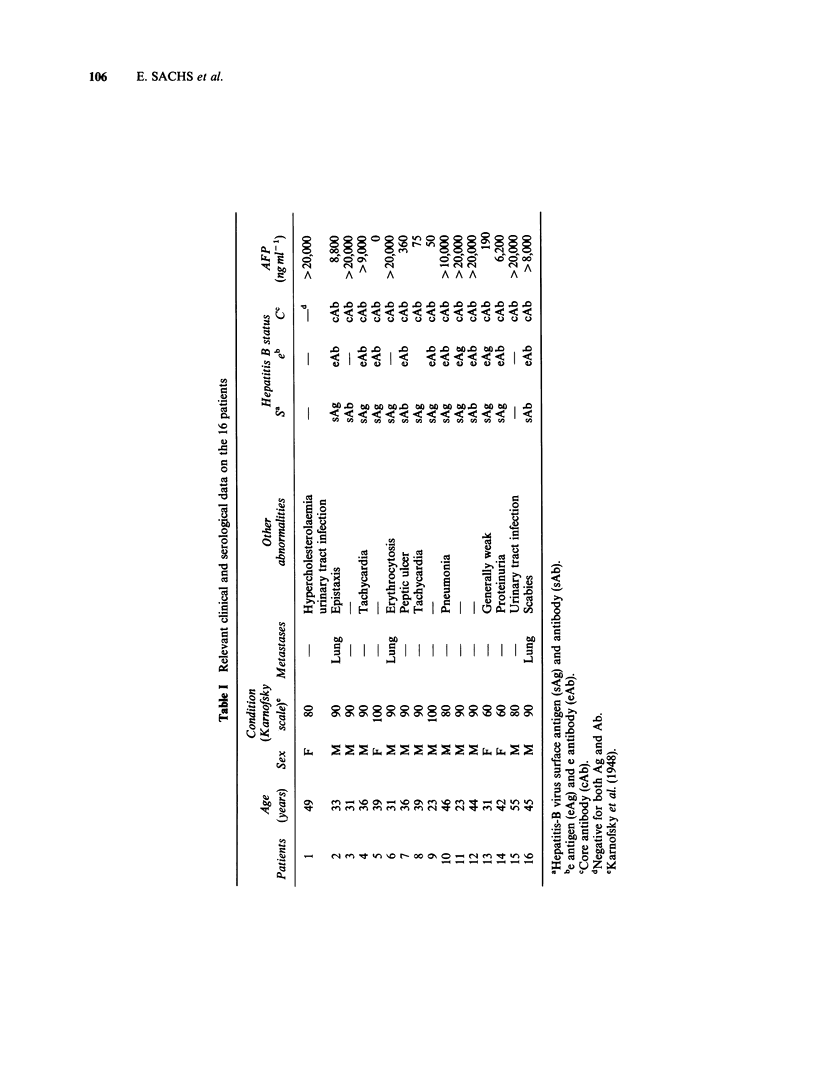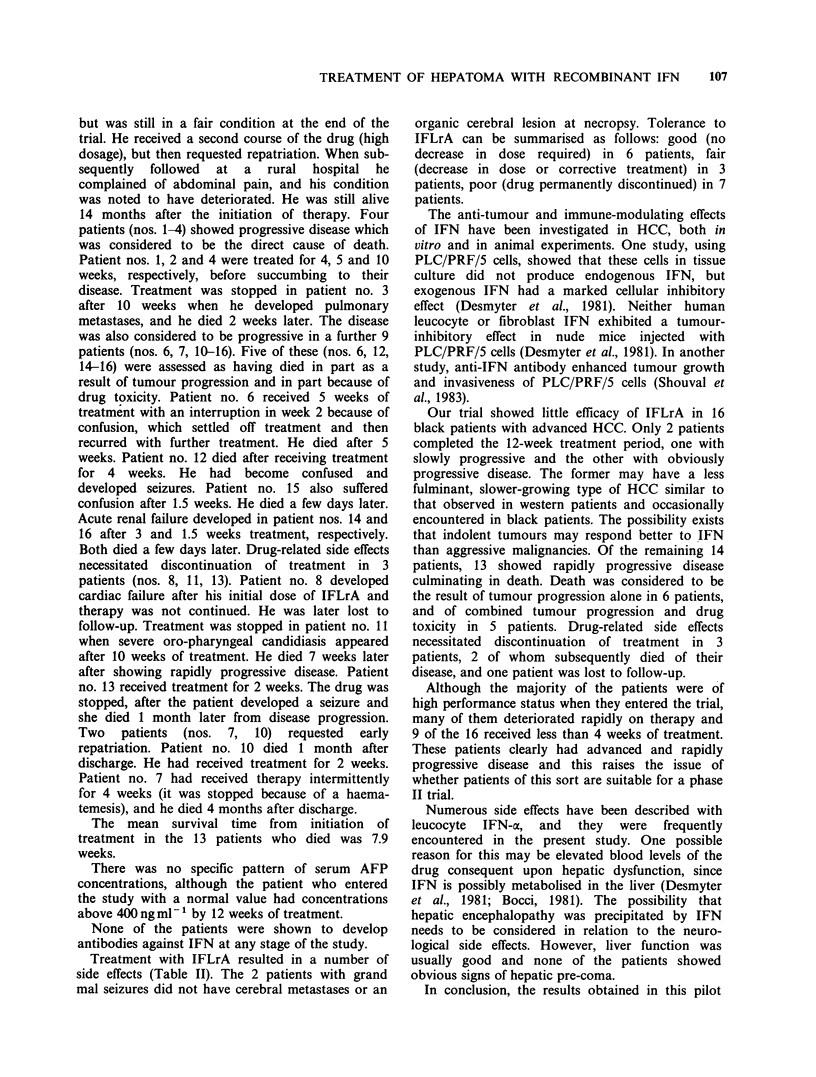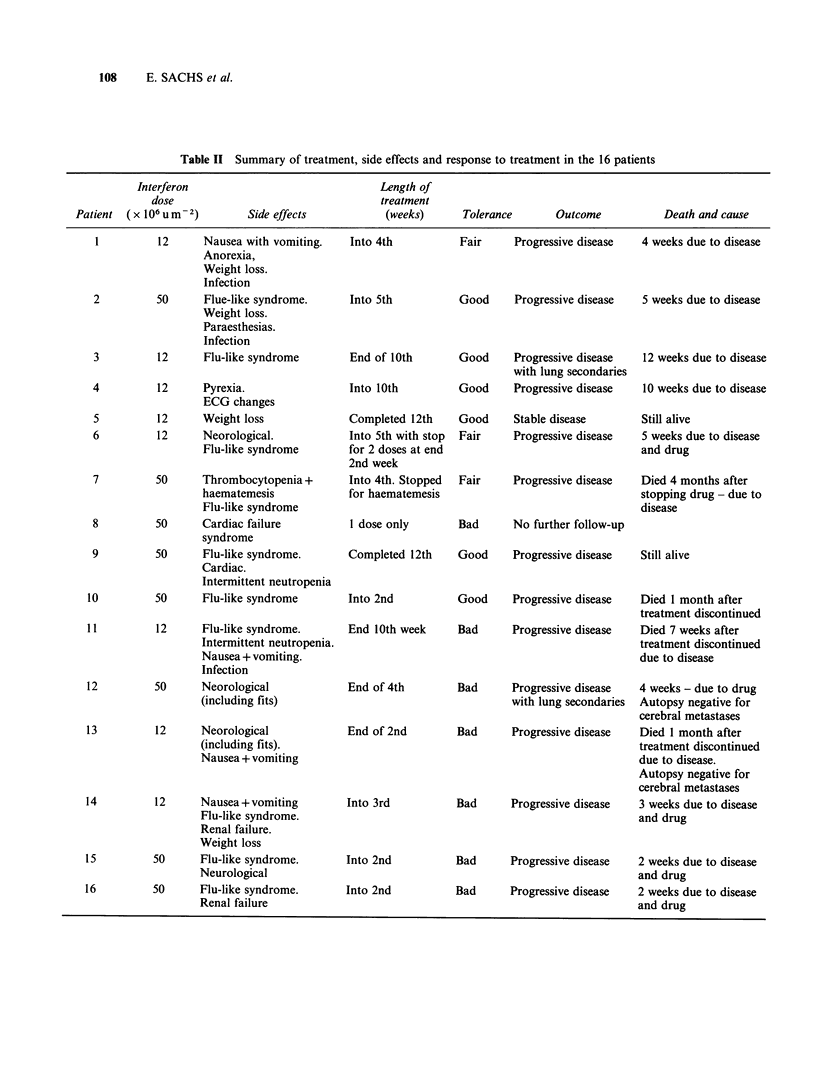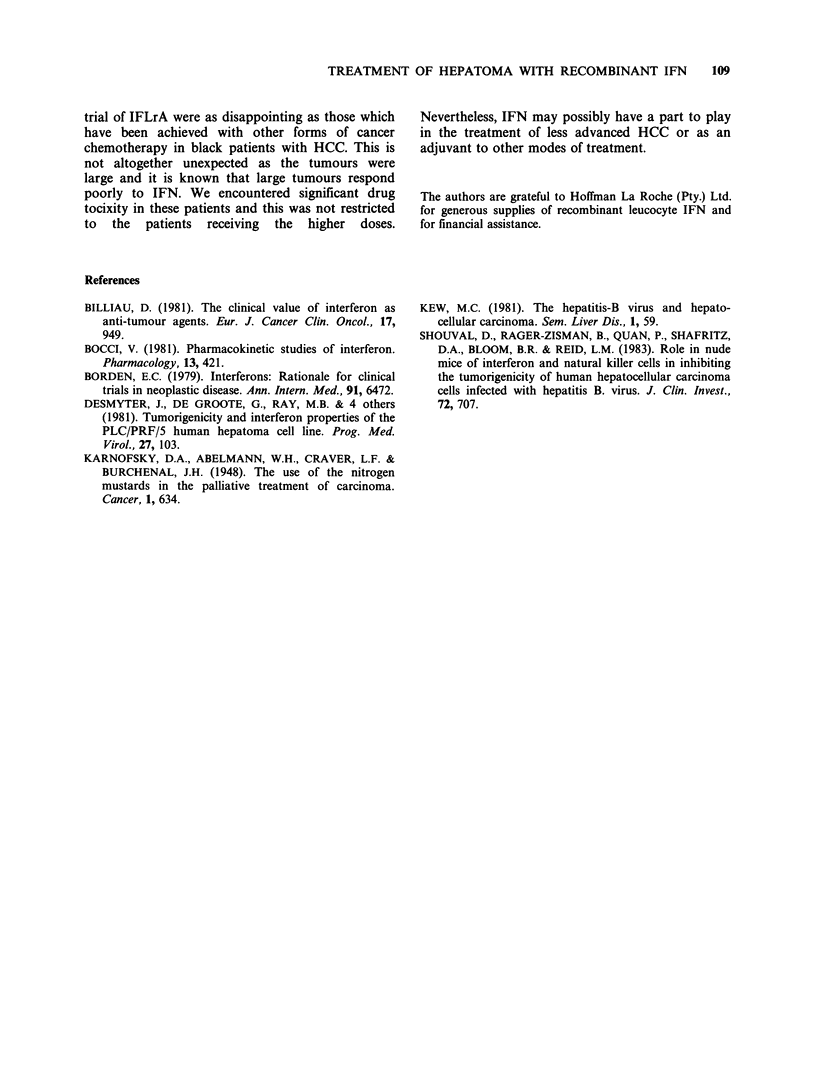# Treatment of hepatocellular carcinoma with recombinant leucocyte interferon: a pilot study.

**DOI:** 10.1038/bjc.1985.156

**Published:** 1985-07

**Authors:** E. Sachs, A. M. Di Bisceglie, G. M. Dusheiko, E. Song, S. F. Lyons, B. D. Schoub, M. C. Kew


*Br. J. Cancer* (1985), **52**, 105–109

### Short Communication

### Treatment of hepatocellular carcinoma with recombinant leucocyte interferon: A pilot study

E. Sachs, A.M. Di Bisceglie, G.M. Dusheiko, E. Song, S.F. Lyons, B.D. Schoub & M.C. Kew

*Department of Medicine, University of the Witwatersrand Medical School and Johannesburg and Hillbrow Hospitals, and the National Institute of Virology, Johannesburg, South Africa*

The interferons (IFNs) are glycoproteins which have, in addition to anti-viral activity, anti-proliferative and immunomodulating properties (Borden, 1979; Billiau, 1981). The latter two suggested an anti-tumour potential for these substances and IFN has now been used in the treatment of a variety of malignant diseases. To date, there have been no reports of its use in human hepatocellular carcinoma (HCC). There is considerable epidemiological, serological and molecular biological evidence implicating the hepatitis-B virus in the aetiology of HCC (Kew, 1981), and this provides an additional reason for investigating the effects of IFN on the course of this tumour. We report a pilot trial of recombinant leucocyte alpha IFN (IFLrA; Hoffman la Roche) in the treatment of HCC.

Sixteen patients with inoperable HCC were entered in the trial (Table I). With one exception, all were histologically proved. The exception (no. 16) was considered to have HCC on the basis of the clinical findings and a serum alpha-foetoprotein (AFP) concentration of $8 \times 10^3$ ng ml$^{-1}$ (normal $<10$). Five patients had a well-differentiated, 8 a moderately-differentiated, and 2 a poorly-differentiated tumour. A trabecular pattern was present in 9 patients, a solid pattern in 3 and a scirrhous pattern in 3. One patient had a clear cell variant. The disease was confined to the liver in 13 patients and 3 had pulmonary metastases. All the patients conformed to required inclusion criteria with respect to haematological function (haemoglobin $>10$ g dl$^{-1}$, leucocyte count $>3 \times 10^9$ l$^{-1}$, granulocytes $>10^9$ l$^{-1}$, platelets $>30 \times 10^9$ l$^{-1}$) and renal function (blood urea and creatinine levels $<2.5$ times upper limit of normal). Although hepatic function tests were abnormal, impairment was not severe enough to exclude these patients from the trial (prothrombin ratio $>2.5$ times normal was considered to indicate severe hepatic impairment). None of the patients had a history of, or showed obvious evidence for, cardiac or neurological disease. Some had other diseases or abnormalities. No patient had received prior radiotherapy or chemotherapy. Ten patients showed markers of current hepatitis B virus infection and 5 markers of past infection.

Patients were randomised into two treatment groups: Eight received $12 \times 10^6$ units IFLrA m$^{-2}$ body surface area by i.m. injection 3 times per week (low dosage) and 8 $50 \times 10^6$ units m$^{-2}$ i.m. 3 times per week (high dosage). The drug was administered for up to 12 weeks. The purpose and nature of the trial was explained to the participants and their consent obtained. The trial was approved by the Therapeutics Committee of the Hillbrow Hospital where it was conducted. Dosage reductions or interruptions were made when necessary according to a prescribed protocol. Blood transfusions were administered when haemoglobin levels dropped below 10 g dl$^{-1}$ in 4 patients (nos. 1, 2, 11, 13), and after a haematemesis in one patient (no. 7). All patients received analgesics as necessary and paracetamol for influenza-like symptoms associated with IFN administration. Other appropriate medications were given as required. No aspirin, non-steroidal anti-inflammatory agents or corticosteroids were permitted.

A complete remission was defined as disappearance of all evidence of tumour; regression as a definite decrease in tumour size determined by two investigators; stable disease as no definite increase or decrease in tumour size assessed by two investigators; and progression as a definite increase in tumour size or the appearance of a new lesion agreed by two investigators.

Only 2 patients (nos. 5, 9) completed the 12 week course of treatment (Table II). In patient no. 5 the disease remained stable during this period, and she has since received 4 further courses of IFLrA at the same (low) dosage. The disease was slowly progressive for 1 year after therapy was initiated but in the past few months her condition has deteriorated markedly and she has become deeply jaundiced. Patient no. 9 showed progressive disease,

Correspondence: M.C. Kew.

Received 11 December 1984; and in revised form 25 March 1985.

© The Macmillan Press Ltd., 1985

**Table I** Relevant clinical and serological data on the 16 patients

| *Patients* | *Age (years)* | *Sex* | *Condition (Karnofsky scale)*[e] | *Metastases* | *Other abnormalities* | *Hepatitis B status* $S^a$ | $e^b$ | $C^c$ | *AFP* $(ng\,ml^{-1})$ |
|---|---|---|---|---|---|---|---|---|---|
| 1 | 49 | F | 80 | — | Hypercholesterolaemia urinary tract infection | — | — | —[d] | >20,000 |
| 2 | 33 | M | 90 | Lung | Epistaxis | sAg | eAb | cAb | 8,800 |
| 3 | 31 | M | 90 | — | — | sAb | — | cAb | >20,000 |
| 4 | 36 | M | 90 | — | Tachycardia | sAg | eAb | cAb | >9,000 |
| 5 | 39 | F | 100 | — | — | sAg | eAb | cAb | 0 |
| 6 | 31 | M | 90 | Lung | Erythrocytosis | sAg | — | cAb | >20,000 |
| 7 | 36 | M | 90 | — | Peptic ulcer | sAb | eAb | cAb | 360 |
| 8 | 39 | M | 90 | — | Tachycardia | sAg | | cAb | 75 |
| 9 | 23 | M | 100 | — | — | sAg | eAb | cAb | 50 |
| 10 | 46 | M | 80 | — | Pneumonia | sAg | eAb | cAb | >10,000 |
| 11 | 23 | M | 90 | — | — | sAg | eAg | cAb | >20,000 |
| 12 | 44 | M | 90 | — | — | sAb | eAb | cAb | >20,000 |
| 13 | 31 | F | 60 | — | Generally weak | sAg | eAg | cAb | 190 |
| 14 | 42 | F | 60 | — | Proteinuria | sAg | eAb | cAb | 6,200 |
| 15 | 55 | M | 80 | — | Urinary tract infection | — | — | cAb | >20,000 |
| 16 | 45 | M | 90 | Lung | Scabies | sAb | eAb | cAb | >8,000 |

[a]Hepatitis-B virus surface antigen (sAg) and antibody (sAb).

[b]e antigen (eAg) and e antibody (eAb).

[c]Core antibody (cAb).

[d]Negative for both Ag and Ab.

[e]Karnofsky *et al.* (1948).

but was still in a fair condition at the end of the trial. He received a second course of the drug (high dosage), but then requested repatriation. When subsequently followed at a rural hospital he complained of abdominal pain, and his condition was noted to have deteriorated. He was still alive 14 months after the initiation of therapy. Four patients (nos. 1–4) showed progressive disease which was considered to be the direct cause of death. Patient nos. 1, 2 and 4 were treated for 4, 5 and 10 weeks, respectively, before succumbing to their disease. Treatment was stopped in patient no. 3 after 10 weeks when he developed pulmonary metastases, and he died 2 weeks later. The disease was also considered to be progressive in a further 9 patients (nos. 6, 7, 10–16). Five of these (nos. 6, 12, 14–16) were assessed as having died in part as a result of tumour progression and in part because of drug toxicity. Patient no. 6 received 5 weeks of treatment with an interruption in week 2 because of confusion, which settled off treatment and then recurred with further treatment. He died after 5 weeks. Patient no. 12 died after receiving treatment for 4 weeks. He had become confused and developed seizures. Patient no. 15 also suffered confusion after 1.5 weeks. He died a few days later. Acute renal failure developed in patient nos. 14 and 16 after 3 and 1.5 weeks treatment, respectively. Both died a few days later. Drug-related side effects necessitated discontinuation of treatment in 3 patients (nos. 8, 11, 13). Patient no. 8 developed cardiac failure after his initial dose of IFLrA and therapy was not continued. He was later lost to follow-up. Treatment was stopped in patient no. 11 when severe oro-pharyngeal candidiasis appeared after 10 weeks of treatment. He died 7 weeks later after showing rapidly progressive disease. Patient no. 13 received treatment for 2 weeks. The drug was stopped, after the patient developed a seizure and she died 1 month later from disease progression. Two patients (nos. 7, 10) requested early repatriation. Patient no. 10 died 1 month after discharge. He had received treatment for 2 weeks. Patient no. 7 had received therapy intermittently for 4 weeks (it was stopped because of a haematemesis), and he died 4 months after discharge.

The mean survival time from initiation of treatment in the 13 patients who died was 7.9 weeks.

There was no specific pattern of serum AFP concentrations, although the patient who entered the study with a normal value had concentrations above 400 ng ml$^{-1}$ by 12 weeks of treatment.

None of the patients were shown to develop antibodies against IFN at any stage of the study.

Treatment with IFLrA resulted in a number of side effects (Table II). The 2 patients with grand mal seizures did not have cerebral metastases or an organic cerebral lesion at necropsy. Tolerance to IFLrA can be summarised as follows: good (no decrease in dose required) in 6 patients, fair (decrease in dose or corrective treatment) in 3 patients, poor (drug permanently discontinued) in 7 patients.

The anti-tumour and immune-modulating effects of IFN have been investigated in HCC, both *in vitro* and in animal experiments. One study, using PLC/PRF/5 cells, showed that these cells in tissue culture did not produce endogenous IFN, but exogenous IFN had a marked cellular inhibitory effect (Desmyter *et al.*, 1981). Neither human leucocyte or fibroblast IFN exhibited a tumour-inhibitory effect in nude mice injected with PLC/PRF/5 cells (Desmyter *et al.*, 1981). In another study, anti-IFN antibody enhanced tumour growth and invasiveness of PLC/PRF/5 cells (Shouval *et al.*, 1983).

Our trial showed little efficacy of IFLrA in 16 black patients with advanced HCC. Only 2 patients completed the 12-week treatment period, one with slowly progressive and the other with obviously progressive disease. The former may have a less fulminant, slower-growing type of HCC similar to that observed in western patients and occasionally encountered in black patients. The possibility exists that indolent tumours may respond better to IFN than aggressive malignancies. Of the remaining 14 patients, 13 showed rapidly progressive disease culminating in death. Death was considered to be the result of tumour progression alone in 6 patients, and of combined tumour progression and drug toxicity in 5 patients. Drug-related side effects necessitated discontinuation of treatment in 3 patients, 2 of whom subsequently died of their disease, and one patient was lost to follow-up.

Although the majority of the patients were of high performance status when they entered the trial, many of them deteriorated rapidly on therapy and 9 of the 16 received less than 4 weeks of treatment. These patients clearly had advanced and rapidly progressive disease and this raises the issue of whether patients of this sort are suitable for a phase II trial.

Numerous side effects have been described with leucocyte IFN-$\alpha$, and they were frequently encountered in the present study. One possible reason for this may be elevated blood levels of the drug consequent upon hepatic dysfunction, since IFN is possibly metabolised in the liver (Desmyter *et al.*, 1981; Bocci, 1981). The possibility that hepatic encephalopathy was precipitated by IFN needs to be considered in relation to the neurological side effects. However, liver function was usually good and none of the patients showed obvious signs of hepatic pre-coma.

In conclusion, the results obtained in this pilot

**Table II** Summary of treatment, side effects and response to treatment in the 16 patients

| *Patient* | *Interferon dose* ($\times 10^6$ u m$^{-2}$) | *Side effects* | *Length of treatment* (*weeks*) | *Tolerance* | *Outcome* | *Death and cause* |
|---|---|---|---|---|---|---|
| 1 | 12 | Nausea with vomiting. Anorexia, Weight loss. Infection | Into 4th | Fair | Progressive disease | 4 weeks due to disease |
| 2 | 50 | Flue-like syndrome. Weight loss. Paraesthesias. Infection | Into 5th | Good | Progressive disease | 5 weeks due to disease |
| 3 | 12 | Flu-like syndrome | End of 10th | Good | Progressive disease with lung secondaries | 12 weeks due to disease |
| 4 | 12 | Pyrexia. ECG changes | Into 10th | Good | Progressive disease | 10 weeks due to disease |
| 5 | 12 | Weight loss | Completed 12th | Good | Stable disease | Still alive |
| 6 | 12 | Neorological. Flu-like syndrome | Into 5th with stop for 2 doses at end 2nd week | Fair | Progressive disease | 5 weeks due to disease and drug |
| 7 | 50 | Thrombocytopenia + haematemesis Flu-like syndrome | Into 4th. Stopped for haematemesis | Fair | Progressive disease | Died 4 months after stopping drug – due to disease |
| 8 | 50 | Cardiac failure syndrome | 1 dose only | Bad | No further follow-up | |
| 9 | 50 | Flu-like syndrome. Cardiac. Intermittent neutropenia | Completed 12th | Good | Progressive disease | Still alive |
| 10 | 50 | Flu-like syndrome | Into 2nd | Good | Progressive disease | Died 1 month after treatment discontinued |
| 11 | 12 | Flu-like syndrome. Intermittent neutropenia. Nausea + vomiting. Infection | End 10th week | Bad | Progressive disease | Died 7 weeks after treatment discontinued due to disease |
| 12 | 50 | Neorological (including fits) | End of 4th | Bad | Progressive disease with lung secondaries | 4 weeks – due to drug Autopsy negative for cerebral metastases |
| 13 | 12 | Neorological (including fits). Nausea + vomiting | End of 2nd | Bad | Progressive disease | Died 1 month after treatment discontinued due to disease. Autopsy negative for cerebral metastases |
| 14 | 12 | Nausea + vomiting Flu-like syndrome. Renal failure. Weight loss | Into 3rd | Bad | Progressive disease | 3 weeks due to disease and drug |
| 15 | 50 | Flu-like syndrome. Neurological | Into 2nd | Bad | Progressive disease | 2 weeks due to disease and drug |
| 16 | 50 | Flu-like syndrome. Renal failure | Into 2nd | Bad | Progressive disease | 2 weeks due to disease and drug |

trial of IFLrA were as disappointing as those which have been achieved with other forms of cancer chemotherapy in black patients with HCC. This is not altogether unexpected as the tumours were large and it is known that large tumours respond poorly to IFN. We encountered significant drug tocixity in these patients and this was not restricted to the patients receiving the higher doses. Nevertheless, IFN may possibly have a part to play in the treatment of less advanced HCC or as an adjuvant to other modes of treatment.

The authors are grateful to Hoffman La Roche (Pty.) Ltd. for generous supplies of recombinant leucocyte IFN and for financial assistance.

### References

BILLIAU, D. (1981). The clinical value of interferon as anti-tumour agents. *Eur. J. Cancer Clin. Oncol.*, **17**, 949.

BOCCI, V. (1981). Pharmacokinetic studies of interferon. *Pharmacology*, **13**, 421.

BORDEN, E.C. (1979). Interferons: Rationale for clinical trials in neoplastic disease. *Ann. Intern. Med.*, **91**, 6472.

DESMYTER, J., DE GROOTE, G., RAY, M.B. & 4 others (1981). Tumorigenicity and interferon properties of the PLC/PRF/5 human hepatoma cell line. *Prog. Med. Virol.*, **27**, 103.

KARNOFSKY, D.A., ABELMANN, W.H., CRAVER, L.F. & BURCHENAL, J.H. (1948). The use of the nitrogen mustards in the palliative treatment of carcinoma. *Cancer*, **1**, 634.

KEW, M.C. (1981). The hepatitis-B virus and hepatocellular carcinoma. *Sem. Liver Dis.*, **1**, 59.

SHOUVAL, D., RAGER-ZISMAN, B., QUAN, P., SHAFRITZ, D.A., BLOOM, B.R. & REID, L.M. (1983). Role in nude mice of interferon and natural killer cells in inhibiting the tumorigenicity of human hepatocellular carcinoma cells infected with hepatitis B. virus. *J. Clin. Invest.*, **72**, 707.